# Treatment of Baldness and Falling out of the Hair

**Published:** 1846-04

**Authors:** 


7. "*Treatment of Baldness and Falling out of the Hair.*—Dr. Wilson recommends for falling out and loosening of the hair, to immerse the head in cold water, morning and night, to dry the hair thoroughly, and then bursh the scalp until a warm glow is produced. In women with long hair, the scalp is to be brushed until redness and a warm glow are produced, then wet the roots of the hair with one of the following lotions: I. ℞. Vinegar of cantharides ℥ ss., Eau de Cologne ʒ ij., rose water ℥ j., M.; or II. Eau de Cologne ℥ij., tinct. cantharides ℥ ss., oil of nutmegs ʒ ss., ol. lavender, ten drops, M.; III. ℞. Mezerion bark ℥ j., horse-radish root ℥ j., boiling distilled vinegar, Oss. Let it stand for a week and strain. If the lotion produce smarting or tenderness, the brush may be laid aside, but if no sensation is occasioned, the brushing should be resumed, and a second application of the lotion. This treatment should be practised once or twice a day, or at intervals of a few days, according to the state of the scalp; namely, if tender, less; if insensible, more frequently. The same treatment will prove successful in *baldness;* which, if it happen in patches, the skin should be well brushed with a soft tooth-brush, dipped in distilled vinegar, morning and evening. If either of the above lotions proves too irritating to the skin, use it in smaller quantity and less frequently. No. III. may be diluted with more distilled vinegar. Oil should be used to keep the skin soft and pliant."—*On Healthy Skin, p.* 257. in *Ibid.*

